# Determinants of fluconazole resistance and echinocandin tolerance in *C. parapsilosis* isolates causing a large clonal candidemia outbreak among COVID-19 patients in a Brazilian ICU

**DOI:** 10.1080/22221751.2022.2117093

**Published:** 2022-09-27

**Authors:** Farnaz Daneshnia, João N. de Almeida Júnior, Amir Arastehfar, Lisa Lombardi, Erika Shor, Lis Moreno, Ana Verena Mendes, Maria Goreth Barberino, Danilo Thomaz Yamamoto, Geraldine Butler, David S. Perlin, Arnaldo Lopes Colombo

**Affiliations:** a Center for Discovery and Innovation, Hackensack Meridian Health, Nutley, NJ, USA; b Institute for Biodiversity and Ecosystem Dynamics, University of Amsterdam, Amsterdam, The Netherlands; c Special Mycology Laboratory, Federal University of São Paulo, São Paulo, Brazil; d Clinical Laboratory, Hospital Israelita Albert Einstein, São Paulo, Brazil; e School of Biomolecular and Biomedical Science, Conway Institute, University College Dublin, Dublin, Ireland; f Hackensack Meridian School of Medicine, Nutley, NJ, USA; g Hospital São Rafael, Salvador, Brazil; h Instituto D’OR de Pesquisa e Ensino (IDOR); i Mycology Laboratory, Instituto de Medicina Tropical, Universidade de São Paulo, São Paulo, Brazil; j Georgetown University Lombardi Comprehensive Cancer Center, Washington, DC, USA; k Department of Medicine, Division of Infectious Diseases, Escola Paulista de Medicina, Universidade Federal de São Paulo, São Paulo, Brazil

**Keywords:** *Candida parapsilosis*, outbreak, candidemia, fluconazole resistance, echinocandin tolerance

## Abstract

Patients presenting with severe COVID-19 are predisposed to acquire secondary fungal infections such as COVID-19-associated candidemia (CAC), which are associated with poor clinical outcomes despite antifungal treatment. The extreme burden imposed on clinical facilities during the COVID-19 pandemic has provided a permissive environment for the emergence of clonal outbreaks of multiple *Candida* species, including *C. auris* and *C. parapsilosis*. Here we report the largest clonal CAC outbreak to date caused by fluconazole resistant (FLZR) and echinocandin tolerant (ECT) *C. parapsilosis*. Sixty *C. parapsilosis* strains were obtained from 57 patients at a tertiary care hospital in Brazil, 90% of them were FLZR and ECT. Although only 35.8% of FLZR isolates contained an *ERG11* mutation, all of them contained the *TAC1^L518F^* mutation and significantly overexpressed *CDR1*. Introduction of *TAC1^L518F^* into a susceptible background increased the MIC of fluconazole and voriconazole 8-fold and resulted in significant basal overexpression of *CDR1*. Additionally, FLZR isolates exclusively harboured E1939G outside of Fks1 hotspot-2, which did not confer echinocandin resistance, but significantly increased ECT. Multilocus microsatellite typing showed that 51/60 (85%) of the FLZR isolates belonged to the same cluster, while the susceptible isolates each represented a distinct lineage. Finally, biofilm production in FLZR isolates was significantly lower than in susceptible counterparts Suggesting that it may not be an outbreak determinant. In summary, we show that *TAC1^L518F^* and *FKS1^E1393G^* confer FLZR and ECT, respectively, in CAC-associated *C. parapsilosis*. Our study underscores the importance of antifungal stewardship and effective infection control strategies to mitigate clonal *C. parapsilosis* outbreaks.

## Introduction

The global pandemic of COVID-19 during the last two years has had a profound impact on healthcare settings and predisposed a significant number of patients to develop secondary bacterial and fungal infections [1]. COVID-19-associated candidemia (CAC) is one of the most frequently observed fungal infections complicating COVID-19 [1]. Although the mortality rates among the COVID-19 patients admitted to ICUs are notably high, development of CAC further significantly increases these mortality rates [2]. More importantly, the limited availability of personal protective equipment and the crowdedness of hospital units have created a permissive environment for emergence of outbreaks due to *Candida auris* [3,4] and *C. parapsilosis* [5,6]. Indeed, a recent study from Brazil documented a large outbreak of fluconazole resistant *C. parapsilosis* (FLZR-CP) isolates involving 30 patients in a cardiology ward, which continued despite the application of ethanol-based disinfectant [6]. Similarly, persistence of such outbreaks even after extensive application of quaternary ammonium-based disinfectant has also been reported prior to COVID-19 pandemic [7]. Because FLZR-CP isolates are associated with significantly higher mortality, the emergence of such outbreaks could lead to poorer clinical outcomes [8,9]. In fact, the persistence of such infections has been a motive behind the change in clinical practice and replacement of fluconazole with echinocandins for patients infected with *C. parapsilosis* in centres dealing with clonal FLZR-CP outbreaks, which in turn is leading to the emergence of multidrug-resistant *C. parapsilosis* isolates [10]. The extensive use of antibiotics and antifungals during COVID-19 may also contribute to worsening the problem of the antimicrobial resistance in the aftermath of the pandemic [11,12]. In this scenario, identification of the source of infection in conjunction with effective infection control strategies and antifungal stewardship are instrumental in lowering the risk of antifungal resistance.

Although fluconazole resistance in *C. parapsilosis* is primarily mediated by *ERG11* mutations affecting the binding of drug to its target, such as Y132F and K143R, other factors, including overexpression of efflux pumps (*CDR1* and *MDR1*) and of *ERG11* have been reported among FLZR-CP isolates [13–15]. Such overexpression is mainly driven by gain-of-function (GOF) mutations in transcription factors regulating *CDR1*, *MDR1*, and *ERG11*, namely *TAC1, MRR1*, and *UPC2* [16]. Nonetheless, the potential direct contribution of such GOF mutations to the overexpression of their target genes is still poorly explored in *C. parapsilosis* [17]. Although echinocandin resistance (ECR) is less frequently encountered than FLZR in *C. parapsilosis*, a recent study identified R658G in the Fks1 hotspot 1 (HS1) of MDR in *C. parapsilosis* isolates [10], while other studies have identified either *FKS* mutation outside of the HS region [18] or no *FKS* mutations at all [19]. Although less studied in *Candida* species compared to pathogenic bacterial species, the concept of antifungal tolerance is increasingly being encountered in Medical Mycology, which has the potential to negatively impact therapeutic success, as well as pave the way for emergence of stable antifungal resistance [20,21]. Antifungal tolerance is defined as reduced *in vitro* susceptibility in the absence of known resistance mechanisms, and measurement strategies vary depending on the killing dynamic, where azole tolerance is defined as slow growth above the minimum inhibitory concentrations (MIC) after 48 h using E-test and broth microdilution assays [21]. Echinocandin tolerance, however, quantitatively measures the survival rate using colony forming unit (CFU) at any given time (arbitrary but typically up to 24 h) [20].

The advent of novel precise genetic tools, such as CRISPR-Cas9, has remarkably increased our understanding of fungal pathogenesis and antifungal resistance [22]. For instance, a recent study successfully employed this technique to confirm that Erg11-G458S, but not Erg11-L376I, confers azole resistance in *C. orthopsilosis*, a sibling species of *C. parapsilosis* [23]. However, such tools have not yet been broadly employed to dissect the role of specific mutations in antifungal drug resistance in *C. parapsilosis*.

Herein, we describe the largest to date clonal fungal outbreak in COVID-19 patients due to FLZR and echinocandin tolerant (ECT) *C. parapsilosis* in a single referral hospital in Salvador, Brazil. We also use the CRISPR-Cas9 technology in *C. parapsilosis* to further our understanding of fluconazole resistance and echinocandin tolerance in this fungal pathogen. Collectively, our study cautions against the extensive use of antifungal drugs in severely ill COVID-19 patients and suggests that the implementation of strict antifungal stewardship and effective infection control strategies are required to prevent the occurrence of antifungal drug-resistant fungal outbreaks. Importantly, our study also advocates for *FKS* sequencing even among susceptible *Candida* isolates, showing that mutations outside the canonical HS regions should not be overlooked.

## Methods

### Patients, isolate collection, and identification

Severely ill COVID-19 patients referred to São Rafael hospital located in Salvador, Brazil, who presented with candidemia due to *C. parapsislosis* were recruited to the current study. Candidemia was defined when *C. parapsilosis* was recovered from blood samples. Our hospital has 329 beds and gives care to adult and paediatric patients, and it was also one of the major referral centres during the COVID-19 pandemic, with an admission rate of 1315 and 1644 patients during 2020 and 2021, respectively. *C. parapsilosis* isolates recovered from the blood samples of patients placed in a COVID-19 ICU were identified by ITS1 and ITS4 primers as described previously [24]. Any *C. parapsilosis* isolates recovered from the blood samples of COVID-19 patients, including sequential isolates, were included and investigated in the current study. This study was approved by local ethical committee of our centre (5.412.257).

### Antifungal susceptibility testing (AFST)

AFST used the broth microdilution of CLSI M27/A3 protocol [25]. Fluconazole, voriconazole, Amphotericin B (AMB) (all from Sigma-Aldrich, St. Louis, MO, United States), micafungin, anidulafungin (both from Pfizer, New York, NY, United States) were included. MICs were assessed visually after 24 h incubation at 37°C. Isolates with MIC≥ 8 µg/ml were considered as fluconazole, micafungin, and anidulafungin resistant, while those with MIC≥ 1 µg/ml were defined as voriconazole resistant [26].

### Multi-locus microsatellite typing (MLMT)

*C. parapsilosis sensu stricto* isolates and the reference strain, ATCC 22019, were subjected to a previously described MLMT approach [27], which PCR amplified eight different loci. After separation on 3% agarose gel, PCR products were stained with GelRed™ (Biotium, Fremont, CA, USA), and visualized with the UVITEC gel documentation system (Cleaver Scientific, Rugby, Warks, UK). Dice coefficient was used to examine the allelic profiles and Bionumerics software v. 7.6 (Applied Maths, Sint-Martens-Latem, Belgium) was used for clustering using unweighted pair group method with arithmetic mean (UPGMA) employing the. Cluster was defined, when ≥2 isolates showed an identical allelic profile [28,29].

### Analysis of biofilm production

To assess biofilm formation, we used a previously described protocol [30] with a few modifications. Briefly, *C. parapsilosis* isolates were grown on YPD-agar overnight at 37°C and a single colony was inoculated into 5 ml YPD broth and incubated at 37°C for overnight (150 rpm). The next day, the OD_600 nm_ of the isolates was adjusted to 1 in YPD broth, and 200 µl from each culture (in triplicate) were transferred to a 96-well microtiter plate and incubated at 37°C for 24 without shaking. After 24 h, the nonadherent cells were removed by washing with distilled water three times, and after air-drying the biofilms, 100 µl of 0.1% (w/v) crystal violet were added to each well and incubated at 37°C for 30 min. Subsequently, the plates were washed three times with distilled water, air-dried, and 200 µl of a solution containing 1% (w/v) SDS and 50% ethanol was added to release the biofilms. Finally, using a plate reader (Infinite®PRO, TECAN) the crystal violet absorbance was measured at OD_490nm_. The biofilm formation for each isolate was measured using two biological replicates in triplicate.

### Sequencing

PCR amplification and sequencing of *ERG11*, HS1 and HS2 of *FKS1*, *TAC1, UPC2,* and *MRR1* were performed as described previously [31]. After assembly and curation of the sequence data, they were aligned against their WT sequences (*ERG11 *= GQ302972, *TAC1 *= HE605204, *MRR1 *= HE605205, *UPC2 *= HE605206, and *FKS1 *= EU221325.1).

### RNA extraction and gene expression analysis

Overnight *C. parapsilosis* cultures (150 rpm and 37°C) were washed with PBS once, and the OD_600nm_ of the cultures was adjusted at 0.5 using fresh YPD, followed by incubation at 37°C and 250 rpm for another 6 h. Upon washing with PBS, *C. parapsilosis* isolates (10^5^ cells/ml), were incubated in RPMI 1640 containing fluconazole one dilution below the minimum inhibitory concentration (MIC) at 37°C and 250 rpm for 90 min. The pellets were then collected by centrifugation (13,000 rpm for 5 min) and stored at −80°C. RNA samples were extracted using a previously described approach [32], subjected to DNase treatment (QIAGEN), and finally repurified using an RNeasy mini-Kit (QIAGEN) as per the manufacturer's suggestion.

qPCR was performed using the primers described previously [33], which included One-Step TB Green PrimeScript RT–PCR Kit II (Perfect Real Time, TaKaRa, Shiga, Japan). qPCRs containing 40 ng of RNA samples, 0.4 µM of primers, 0.8 µl of enzyme and 10 µl of buffer in a final volume of 20 µl were subjected to an Mx3005P qPCR System (Agilent Technologies, Santa Clara, USA).

Experiments were carried out in two biological and at least two technical replicates, and gene expression data were normalized against *ACT1* gene [33]. Fold changes were determined using normalized data of *C. parapsilosis* cells treated with fluconazole relative to untreated initial inoculums of each sample using 2^−ΔΔCT^ as described previously [34]. Overexpression was defined as a fold change ≥2 relative to the untreated cells. Basal expression values for each untreated samples were calculated using the following formula: 2^−ΔCt^, where the ΔCt refers to Ct gene of target minus the Ct *ACT1*.

### Micafungin tolerance

Overnight cultures of *C. parapsilosis* isolates (37°C and 150 rpm) were washed twice with PBS and 50 µl of 2 × 10^8^ cells were inoculated in 1 ml of RPMI1640 containing 4 µg/ml of micafungin. We used this concentration since it differentiates the susceptible from non-susceptible *C. parapsilosis* isolates. Cultures were incubated at 37°C and 150 rpm and plating was performed at each time-points (3, 6, and 24 h). Colony forming units (CFUs) of treated isolates were normalized against untreated positive controls. This experiment involved three biological replicates of two independent *FKS1* mutants carrying G1393E and the wild-type (WT) parental strains.

### Introduction of single nucleotide polymorphisms (SNPs) in the ATCC22019 background using CRISPR-Cas9

We used the pCP-tRNA CRISPR-Cas9 plasmid-based system [35] to introduce mutations in the sequences of *TAC1* and *FKS1* into the *C. parapsilosis* ATCC 22019 background. Suitable protospacer adjacent motif (PAM) sequences targeting *TAC1* and *FKS1* were selected using EuPaGDT [36], based on their specificity and proximity to the desired cut site (*TAC1*-g1 and *FKS1*-g1, see Table 1). Each guide RNA was then generated by annealing of two 23-bp oligonucleotides carrying appropriate overhanging ends and was cloned into the SapI-digested pCP-tRNA plasmid (see Table 1). Each Repair Template (RT; TAC1-RT1 and FKS-RT1, Table 1) was designed to: (i) introduce the desired amino acid change (L518F into Tac1; E1393G into Fks1), and (ii) introduce synonymous SNPs to prevent Cas9 from cutting the edited site by changing the seed sequence [37]. The 100-nt RTs were generated with ExTaq DNA polymerase (TaKaRa Bio, USA) by primer extension from two oligonucleotide primers with 20 bp overlaps at the 3′-ends.

**Table 1.** Oligonucleotides used to generate mutant *C. parapsilosis* isolates carrying *TAC1^L518F^ and FKS1^E1393G^*.

**Production of the guide RNA** The protospacer sequence/PAM site is indicated, followed by the 23-mers (TOP and BOT oligos) for generating the guide RNA. Overhangs are highlighted in bold		
TAC1-g1 (sequence)	5^′^-GTGGCTGATGAGGCATTACT/TGG-3^′^	
TAC1-g1-TOP	5^′^-**CCA**GTGGCTGATGAGGCATTACT-3^′^	Annealing of the 23-mer to produce the guide RNA that was cloned into SapI-digested pCP-tRNA
TAC1-g1-BOT	5^′^-**AAC**AGTAATGCCTCATCAGCCAC-3^′^	
FKS1-g1 (sequence)	5^′^-AGTTGATTGAAAGAGGTGTG/TGG-3^′^	
FKS1-g1-TOP	5^′^-**CCA**AGTTGATTGAAAGAGGTGTG-3^′^	Annealing of the 23-mer to produce the guide RNA that was cloned into SapI-digested pCP-tRNA
FKS1-g1-BOT	5^′^-**AAC**CACACCTCTTTCAATCAACT-3^′^	
**Synthesis of RTs** The sequence of each RT is indicated, followed by the long oligos (TOP and BOT) used for primer extension. The SNPs are highlighted in bold, and described on the right (the numbering refers to the *ORF*); syn = synonymous		
TAC1-RT-1 (sequence)	5^′^-GGCGACGAATTGGATCGTCAAATGTCGATTGCAGTGGCTGATGAGGCATT**GT**TTGGAGATCCAGCACTACCACTCAGTTTTCGATTGTTGAAAAAGTTGA-3^′^	SNPs introduced: A1551G [syn SNP]; C1552T [non syn SNP]
TAC1-RT-1-TOP	5^′^-GGCGACGAATTGGATCGTCAAATGTCGATTGCAGTGGCTGATGAGGCATTGTTTGGAGAT-3^′^	
TAC1-RT-1-BOT	5^′^-TCAACTTTTTCAACAATCGAAAACTGAGTGGTAGTGCTGGATCTCCAAACAATGCCTCAT-3^′^	
FKS1-RT-1 (sequence)	5^′^-TCTTCATTTCGTTCATTCCATTGGTTGTTCAAG**G**GTTGATTGAAAGAGG**A**GTCTGGAAAGCTTGTCAAAGATTTGTTAGACATTTCATTTCGTTGTCACC-3^′^	A4178G [non syn SNP]; G4197C [syn SNP]; T4194A [syn SNP]
FKS1-RT-1-TOP	5^′^-TCTTCATTTCGTTCATTCCATTGGTTGTTCAAGGGTTGATTGAAAGAGGAGTCTGGAAAG-3^′^	
FKS1-RT-1-BOT	5^′^-GGTGACAACGAAATGAAATGTCTAACAAATCTTTGACAAGCTTTCCAGACTCCTCTTTCA-3^′^	
**Sequencing of mutated loci**		
sTAC1-F	5^′^-GGTATGCTCAGGAGATTGGA-3^′^	Sequencing of TAC1 edited site
sTAC1-R	5^′^-ATAGTTCCACGTTCAGGCTC-3^′^	
sFKS1-F	5^′^-CGGACATCCTGGTTTCCATA-3^′^	Sequencing of FKS1 edited site
sFKS1-R	5^′^-CAATGAAGACAACGAAGCCC-3^′^	

Yeast cells were transformed with 5 μg of the relevant plasmid and 25 μl of unpurified RT using the lithium acetate method described in ref. [38], with minor modifications (starting OD_600nm_ of YPD culture 0.1 instead of 0.05). Transformants were plated onto parallel YPD agar plates containing 200 μg/ml nourseothricin (Jena Bioscience GmbH, Germany) and incubated at 30°C for 48 h. Representative mutants were sequenced by Sanger sequencing (MWG/Eurofins). For each mutant strain (LL-TAC1 and LL-FKS1), two independent lineages (A and B) carrying the desired mutations were patched onto YPD agar without selection twice to induce the loss of the pCP-tRNA plasmid. The resulting strains carried the desired mutations and did not contain the Cas9-expressing plasmid anymore.

### Statistical analysis

Excel (Microsoft, Redmond, WA, USA) was used to carry out the statistical analysis. T-test (tow-tailed) was used to define the statistical significance for biofilm formation and echinocandin tolerance. Values < 0.05 were considered as statistically significant.

## Results and discussion

We evaluated a total of 60 isolates cultured from 57 patients who had developed *C. parapsilosis* candidemia in the COVID-19 ICU with three of them had two sequential positive blood bottles. Four (7%) of the patients had previous exposure to fluconazole, while 42 (73.7%) had received an echinocandin before fungemia (median 14 days, interquartile range 9–14 days) (Supplementary Table 1). Most of the patients (*n *= 54, 93%) had a central venous catheter when fungemia was diagnosed, and the 30-day overall mortality rate was 59.6%.

**Figure 1.**
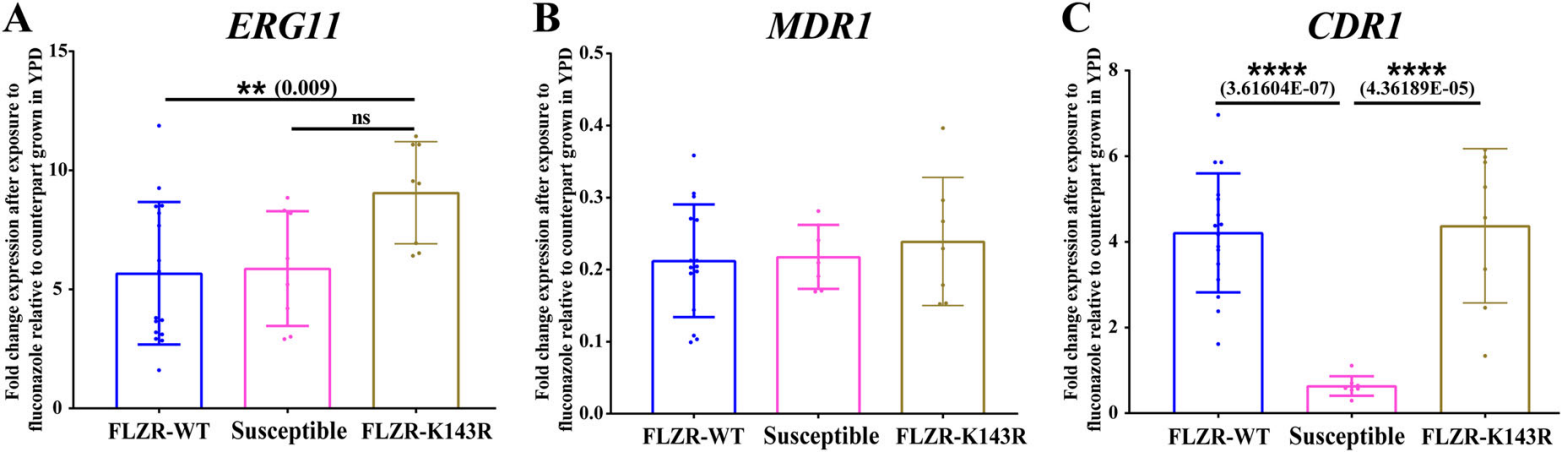
Expression profile of *ERG11* (A), *MDR1* (B), and *CDR1* (C) from a selected number of *C. parapsilosis* isolates (*n *= 8) after exposure to fluconazole, which showed that fluconazole-resistant isolates (FLZR) significantly overexpressed *CDR1* relative to susceptible ones. *C. parapsilosis* isolates grown at logarithmic phase were subjected to one dilution below MIC of fluconazole for 90 min, and after RNA extraction, relative gene expression was assessed as described in methods section.

Our AFST data revealed that 53 of 60 (88.3%) isolates tested were FLZR (≥ 8 µg/ml), which all had intermediate phenotype against voriconazole (Table 2 and supplementary Table 1); while none of the isolates showed resistance to echinocandins and AMB. Although susceptible to both micafungin and anidulafungin, all FLZR isolates had one or two dilution higher MICs compared to FLZS counterparts (Supplementary Table 1). Interestingly, the four patients with prior exposure to fluconazole were infected with FLZR isolates. To delineate the mechanism of FLZR, we first sequenced *ERG11* and to our surprise, only 35.1% of these isolates carried a *ERG11* mutation, K143R, which is a well-known mutation conferring fluconazole resistance in numerous *Candida* species [16]. Of note, the fluconazole MIC of FLZR isolates carrying K143R was >8 µg/ml. Because the vast majority of the FLZR isolates were WT for *ERG11* (FLZR-WT hereafter), we suspected the involvement of GOF mutations in major transcription factors (TFs), i.e. *TAC1, UPC2*, and *MRR1*. Therefore, we selected 16 *C. parapsilosis* isolates, including 10 FLZR without *ERG11* mutation, 3 FLZR carrying K143R and 3 FLZS, and sequenced the aforementioned TFs. Interestingly, all the FLZR isolates sequenced contained a mutation in *TAC1*, L518F, which was missing in reference and susceptible strains, while all the isolates were WT for *UPC2* and *MRR1*. This mutation is located in the middle homology region, which is thought to negatively control the activating domain in the C terminus [39]. Therefore, such mutations could potentially render Tac1 active, which subsequently results in overexpression of genes under the control of this transcription factor, including *CDR1* [39]. Consequently, we suspected that FLZR isolates, but not FLZS counterparts, should overexpress *CDR1* after fluconazole exposure. To test this hypothesis, we selected eight of the sequenced strains, including four FLZR without *ERG11* mutations and two FLZS isolates, exposed them to fluconazole at half the MIC for 90 min and measured the level of expression of *CDR1, ERG11*, and *MDR1* (Table 3). While *ERG11* was highly expressed among both FLZR and FLZS isolates (Figure 1A) and *MDR1* was downregulated in all isolates tested (Figure 1B), only FLZR isolates overexpressed *CDR1* (Figure 1C). On the other hand, the basal expression of *CDR1* was significantly lower in FLZR isolates than in FLZS isolates (Supplementary Figure 1A–C). This observation further reinforced our hypothesis that *TAC1^L518F^* could confer the overexpression of *CDR1* upon exposure to fluconazole and therefore confers fluconazole resistance.

**Table 2.** Minimum inhibitory concentration of antifungal agents used against *Candida parapsilosis* isolates (*n *= 60). The number of isolates for each concentration of a given drug is indicated.

Antifungal agent	Minimum inhibitory concentration (µg/ml)													MIC50	MIC90	GM
	0.016	0.032	0.06	0.12	0.25	0.5	1	2	4	8	16	32	64			
Fluconazole						4	3			53^a^				8	16–32	6.64
Voriconazole	4	1	2		53									0.032	0.5	0.192
Micafungin						3	4	53						4	8	3.52
Anidulafungin						1	6	53						4	8	3.56
Amphotericin B					1	47	12							0.5	1	0.567

^a^ Note that 19 strains carrying K143R in Erg11 and L518F in Tac1 had fluconazole MICs >8 µg/ml.

**Table 3.** The characteristics of *Candida parapsilosis* isolates selected for sequencing and gene expression analysis. The expression profile values of the genes studied are based on the average ± standard deviation.

Strain #	FLZ (µg/ml)	VOR (µg/ml)	MICA (µg/ml)	ANI (µg/ml)	HS2-Fks1	Erg11	*CDR1* expression	*ERG11* expression	*MDR1* expression	Tac1	Upc2	Mrr1
1	8	0.25	2	2	E1393G	WT	2.64 ± 0.94	0.19 ± 0.004	9.32 ± 1.86	L518F	WT	WT
33	16	0.25	2	2	E1393G	WT	5.91 ± 0.80	0.11 ± 0.02	7.16 ± 1.37	L518F	WT	WT
40	8	0.25	2	2	E1393G	WT	4.57 ± 0.39	0.31 ± 0.03	3.25 ± 0.38	L518F	WT	WT
51	8	0.25	2	2	E1393G	WT	3.69 ± 0.53	0.24 ± 0.03	2.95 ± 0.98	L518F	WT	WT
7	8	0.25	2	2	E1393G	K143R	2.93 ± 1.36	0.16 ± 0.01	8.6 ± 2.3	L518F	WT	WT
27	16	0.25	2	2	E1393G	K143R	5.81 ± 0.37	0.29 ± 0.07	9.50 ± 2.2	L518F	WT	WT
10	1	0.03	1	1	WT	WT	0.52 ± 0.16	0.24 ± 0.02	7.91 ± 1.11	WT	WT	WT
20	0.5	0.015	0.5	0.5	WT	WT	0.74 ± 0.25	0.20 ± 0.205	3.83 ± 1.08	WT	WT	WT

FLZ: Fluconazole; VOR: Voriconazole; MICA: Micafungin; ANI: Anidulafungin.

**Figure 2.**
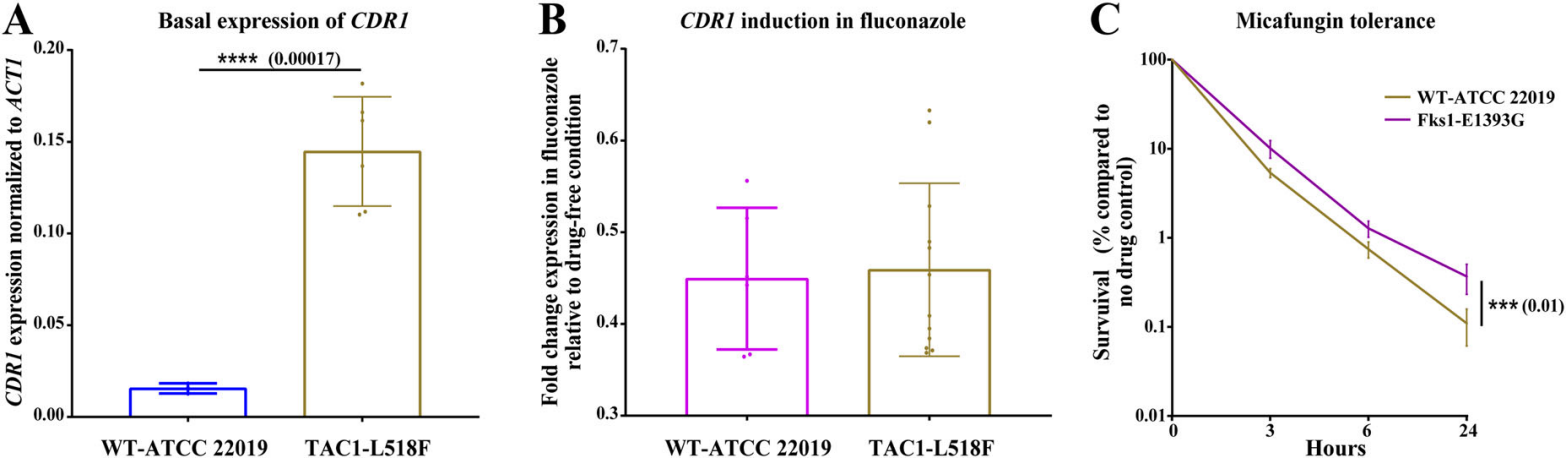
The expression profile of *CDR1* for parental strain ATCC 22019 and its mutants carrying L518F in Tac1 and the micafungin tolerance of ATCC 22019 and it mutant carrying E1393G in Fks1. Mutants carrying *TAC1*^L518F^ (from two independent mutants) had a significantly higher basal expression of *CDR1*, which was not induced upon fluconazole exposure (A and B). Mutants carrying *FKS1*^E1393G^ had a significantly higher tolerance to micafungin (4 µg/ml), which shows the average of two independent mutants.

To ascertain if *TAC1^L518F^* confers fluconazole resistance by inducing *CDR1* overexpression, we used a plasmid-based CRISPR-Cas9 system to introduce this mutation into a susceptible tester strain background, ATCC 22019 [23]. Indeed, the fluconazole and voriconazole MIC of mutants carrying this mutation was increased 8- and 4-fold, 0.5 µg/ml vs 4  µg/ml and 0.03  µg/ml vs 0.125 µg/ml, respectively (Table 4). Unexpectedly, two independent mutants carrying *TAC1^L518F^* showed only basal *CDR1* overexpression (Figure 2A), while fluconazole exposure at three different concentrations (2, 4, and 8 µg/ml) did not cause *CDR1* overexpression (Figure 2B). Therefore, we reasoned that the increase in the basal expression of *CDR1* was sufficient to confer fluconazole resistance. We speculate that basal overexpression of *CDR1* may carry a fitness-cost given that such isolates would constitutively overexpress *CDR1*, which carries a high energy demand. However, given the nutritional immunity imposed by host restrict ATP production, infecting several patients may allow the clinical isolates to overcome this fitness cost by controlling the high basal expression of *CDR1* and only induce overexpression in the presence of azole. This observation deserves deeper investigation, including application of whole-genome sequencing and transcriptomic analysis to unravel the mechanisms underpinning differential expression of *CDR1* in FLZR *C. parapsilosis*.

**Table 4.** The minimum inhibitory concentration of antifungal drugs against the mutants carrying L518F in Tac1 and E1393G in Fks1 and their parental wild-type (WT) strain.

Strain	Phenotype	Fluconazole (µg/ml)	Voriconazole (µg/ml)	Micafungin (µg/ml)
WT	Susceptible	0.5	0.03	2
L518F	Fluconazole non-susceptible	4	0.125	2
E1393G	Micafungin tolerant	0.5	0.03	2

Because our FLZR isolates also had higher echinocandin MICs compared to susceptible isolates, we sought to sequence HS1 and HS2 of *FKS1*. Surprisingly, all the FLZR isolates carried a nonsynonymous mutation outside of the HS2, E1393G (Supplementary Table 1). Interestingly, E1393 is a highly conserved amino acid across a wide range of fungal species ranging from *Saccharomyces cerevisiae* and *Aspergillus fumigatus* to *C. albicans*; therefore, we wondered if this mutation could confer a higher echinocandin MIC once introduced into a susceptible background. Using CRISPR-Cas9, we introduced this mutation into *C. parapsilosis* ATCC 22019, selected two independent mutants carrying this mutation and subjected them to AFST using micafungin and anidulafungin. We found that the mutants and susceptible parental strain showed the same MIC for both echinocandins (Table 4). Bearing in mind that AFST is a qualitative growth/no growth test and that echinocandins are fungicidal in *Candida*, we sought to gain a deeper insight into the impact of E1393G on killing by echinocandins. To this end, we exposed the mutants and the parental strains to 4 µg/ml micafungin, which is an intermediate concentration (i.e. below the MIC of resistant strains but above the sensitivity of the wild type strain), and measured survival using CFU counts at different time-points (3, 6, and 24 h) after drug exposure. Interestingly, we found that the *FKS1^E1393G^* mutants had a significantly increased survival at all time-points, especially at 24 h (Figure 2C). Therefore, although this *FKS1^E1393G^* does not confer echinocandin resistance as clinically defined, it renders isolates carrying this mutation significantly more tolerant to echinocandins, if we define tolerance as the ability to survive better in a given echinocandin concentration. Although such mutations could potentially modulate the echinocandins binding to β-glucan synthase, gaining a deeper understanding on this matter requires RNAseq studies involving both WT and mutants.

As we and others have previously hypothesized [20,21], the higher level of echinocandin tolerance could translate into a higher level of persistent colonization and a greater likelihood of emergence of stable ECR isolates carrying *FKS1* HS mutations during treatment. We propose that due to the prevalence of ECT *FKS1^E1393G^* mutation, the emergence of such ECR isolates in our centre may only be a matter of time, underscoring the importance of using antifungals only when they are necessary. Indeed, the current guidelines advocate using echinocandins as the first line therapy to treat patients with candidemia because they exert fungicidal activity against several species of *Candida*, have a favourable safety profile, and are associated with better survival in a large patient-level quantitative review of randomized clinical trials [40,41]. Considering the inherent reduced susceptibility and the high rate of tolerance to echinocandins observed in our cohort of patients infected by FLZR *C. parapsilosis*, as well as the emerging reports of MDR *C. parapsilosis* isolates [10], lipid formulations of amphotericin B potentially may be a better choice for therapy [42,43]. The establishment of strict antifungal stewardship and appropriate use of antifungals for *Candida* species, especially those causing outbreaks, is of paramount importance to minimize the risk of emergence of antifungal resistance. Importantly, *in vitro* studies have found that caspofungin treatment is associated with the highest mutation frequency in *FKS* compared to micafungin and anidulafungin and the emergence of ECR in *C. glabrata* [44], which may also warn against the high use of this drug in our centre, as its use may further consolidate the prevalence of such tolerant *C. parapsilosis* isolates.

Consistent with the present study, a recent report identified a few mutations outside of *FKS1* HS1 and HS2 in *C. parapsilosis* but the authors only performed AFST and did not introduce this mutation into a sensitive strain to explore its effect on echinocandin-mediated killing quantitatively [18]. Moreover, ECR isolates lacking *FKS1* mutations have also been reported [19], which implicate the involvement of as yet unknown mechanisms underlying echinocandin resistance. Mutations outside of the *FKS* HS regions in *C. glabrata* have been reported to confer ECR and therapeutic failure *in vivo* [45]. The phenomenon of ECR *C. parapsilosis* is becoming more predominant due to the heavy use of echinocandins in routine clinical practice. Collectively, these observations reinforce the importance of *FKS* sequencing even in echinocandin susceptible isolates. Furthermore, the importance of mutations outside of the *FKS* HS may be currently underestimated, and they may have a profound impact on in-host survival of such isolates during echinocandin exposure, facilitating and accelerating the emergence of echinocandin resistance.

**Figure 3.**
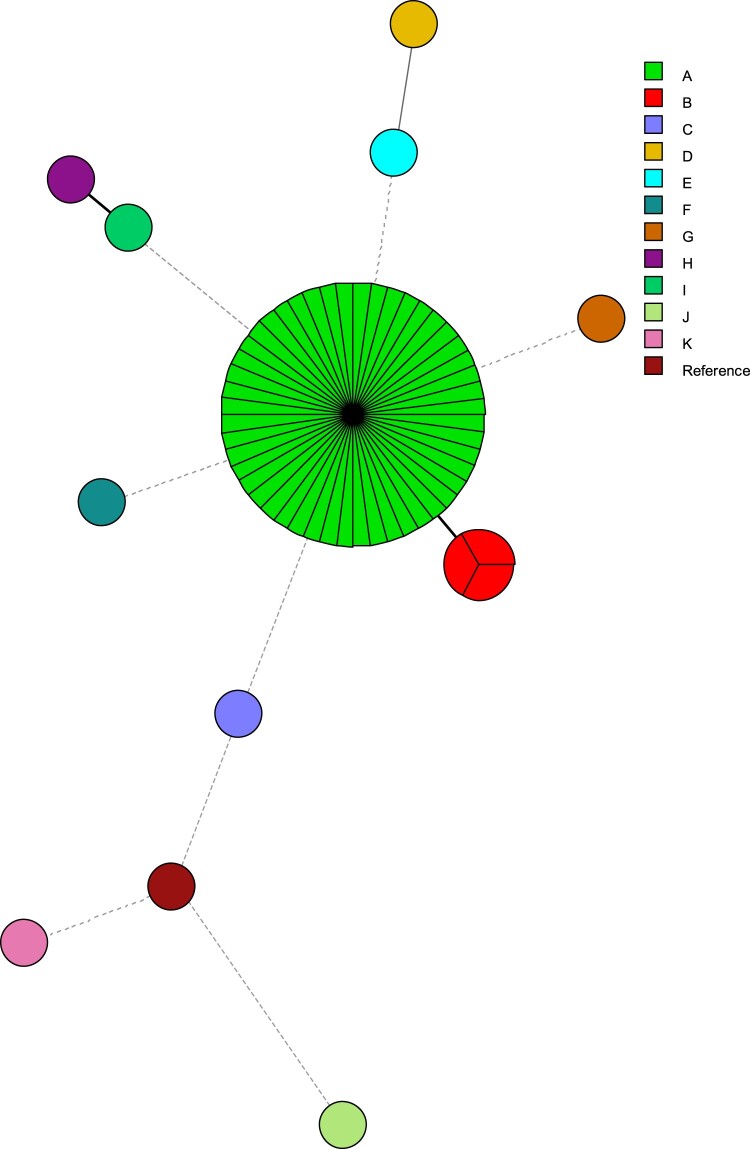
Minimum spanning tree of *C. parapsilosis* isolates.

Given the large number of patients infected over a short period of time and that approximately 90% of the isolates were both FLZR and ECT and harboured the same mutations, we suspected a large clonal outbreak. MLMT was performed, which identified nine minor clusters each represented by a single isolate (Figure 3) and two major clusters close to each other, one containing 80% of the isolates (48/60), and others comprising 2% of the isolates (3/60). Interestingly, each of the fluconazole susceptible isolates had a unique genotype. Therefore, our MLMT findings not only point to a severe clonal outbreak due to FLZR and ECT *C. parapsilosis* isolates, but they also document the largest clonal outbreak due to human fungal pathogens in the context of severely ill COVID-19 patients.

**Figure 4.**
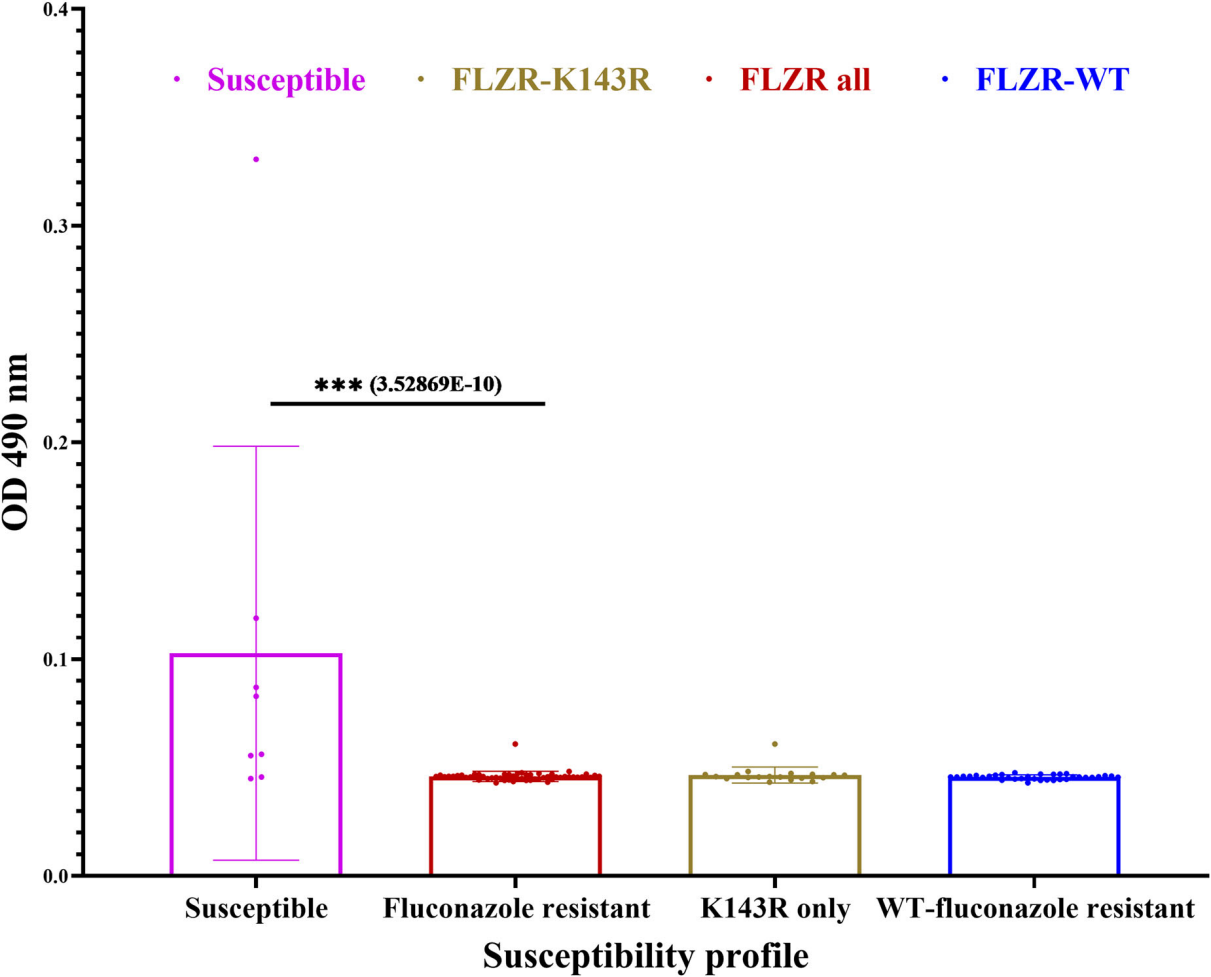
Biofilm formation of fluconazole resistant (FLZR-WT and FLZR-K143R) and fluconazole susceptible isolates. The Y-axis represents biofilm production as a function of absorption at OD490.

Because clonal outbreaks have been associated with increased biofilm production [46], we tested biofilm production of all isolates. To our surprise, none of the FLZR isolates produced biofilms, while the FLZS counterparts produced a higher biofilm level (Figure 4). It should be noted mature biofilm structure is intrinsically resistant to all classes of antifungal drugs, and even immune system, in the absence of genetic changes, whereas the mutation found in our FLZR isolates have been selected for in the presence of fluconazole and only confers protection against azoles in the planktonic conditions. This observation not only challenges the notion that biofilm production is a primary determinant of clonal outbreaks [46] but also questions the findings that biofilms could predict mortality [47]. Indeed, this observation is in line with recent findings obtained with Turkish FLZR *C. parapsilosis* isolates carrying *ERG11^Y132F^*, which did not produce biofilm but were associated with a significantly increased mortality rates in infected patients [8,9]. Since biofilm production is required for survival in hospital environments, such as on abiotic surfaces, we speculate that our outbreak was primarily transferred through skin. Of note, the skin samples for patients recruited in the current study were not available to ascertain this hypothesis. We acknowledge that the in-vitro biofilm formation tested in this study may not fully recapitulate the real-life conditions and therefore the application of the in-vivo catheter model is required to prove this observation.

In conclusion, our study documented the largest clonal outbreak of candidemia due to fluconazole resistant and echinocandin tolerant *C. parapsilosis* isolates among COVID-19 patients, underscoring the importance of rigorous antifungal stewardship to minimize the risk of dangerous outbreaks due to MDR *C. parapsilosis*. Furthermore, our study determined the role of *TAC1^L518F^*and *FKS1^E1393G^* in fluconazole resistance and echinocandin tolerance, respectively, through the application of CRISPR-Cas9 precise genome editing.

## Supplementary Material

Supplemental Material
