# Supplementary figures and images for: Determinants of fluconazole resistance and echinocandin tolerance in C. parapsilosis isolates causing a large clonal candidemia outbreak among COVID-19 patients in a Brazilian ICU
Source: Emerg Microbes Infect. 2022 Sep 27;11(1):2264–74. doi: 10.1080/22221751.2022.2117093 (PMC9542950; doi:10.1080/22221751.2022.2117093)

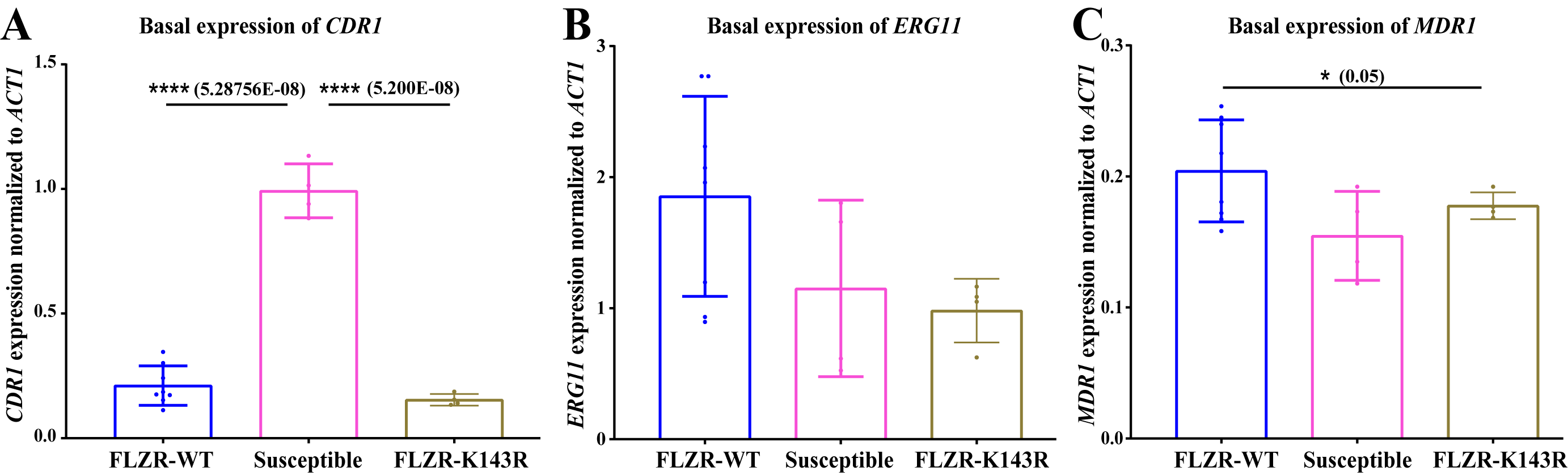

Supplement: Supplemental Material [file TEMI_A_2117093_SM8278.zip › Suppl_Fig_1.tiff]
